# Relations Existing between the Sense of Touch, Etc.

**Published:** 1868-01-15

**Authors:** James T. Newman


### RELATIONS EXISTING BETWEEN THE SENSE OF TOUCH, THE SENSE OF TEMPERATURE, AND THE SENSE OF PAIN.

BY JAMES T. NEWMAN, M. D.

On the 10th of July, 1867, a very singular case came under my observation. Nancy, a negro woman, applied to me for treatment. Upon inquiry into her history, I found that she had been suffering with Hypertrophy of the Heart and persistent Bronchitis. From exposure to cold she became paralyzed, though without loss of consciousness, or deviation of the tongue, when that organ was protruded. The entire right half of the body, including the head, became insensible to pain and to temperature, but there was no loss of motor power. The muscular power, in fact, as measured by the dynamoneter, being greatly increased on the affected side. She could feel the slightest touch on the anæsthetized side, and when the eyes were closed she could discover and pick up a pin from the floor. On washing the hands she could distinctly perceive the shock and movement of the water flowing over them, but was quite unable to distinguish whether it was hot or cold. In winter she could only perceive the temperature with the left half of the body, and the same when standing near the fire. The normal temperature of the skin on the affected side differed so slight as scarcely to be perceptible. But what is more remarkable, neither the pricks of needles nor pinching of the skin could be felt in the least. She suffered from Neuralgia, in the temporal region, at night. In consultation with Dr. C. F. Hart, he advised me to use the galvanic battery; but prior to using the battery I gave the following prescription for the neuralgic pains:

℞.—Quin. Sulph., - - - - - gr. xxx.
Hyoscyami, ext., - - - gr. xxx.

M. and ft. pil. x., divi. part. decem. S.—Take one night and morning.

Upon the exhibition of this medicine I am happy to say she had no more pains. I then commenced to treat her with electricity. I followed up the use of the battery about two months, and, to my great astonishment, she has recovered the use of her limbs, and the sense of feeling is just as good, apparently, as it ever was.

